# Factors associated with deaths in ‘Elderly Housing with Care Services’ in Japan: a cross-sectional study

**DOI:** 10.1186/s12904-017-0241-9

**Published:** 2017-11-23

**Authors:** Kentaro Sugimoto, Yasuko Ogata, Masayo Kashiwagi, Haruka Ueno, Yoshie Yumoto, Yuki Yonekura

**Affiliations:** 1 Graduate School of Health Care Sciences, Tokyo Medical and Dental University, 1-5-45 Yushima, Bunkyo-ku, Tokyo, 113-8510 Japan; 2 Nursing Course, School of Medicine, Yokohama-City University, 3-9 Fukuura, Kanazawa-ku, Yokohama, Kanagawa 236-0004 Japan; 3 Graduate School of Medicine, The University of Tokyo, 7-3-1 Hongo, Bunkyo-ku, Tokyo, 113-8655 Japan; 4 Graduate School of Nursing Science, St. Luke’s International University, 10-1 Akashi-cho, Chuo-ku, Tokyo, 104-0044 Japan

**Keywords:** Aging, Elderly facility, Japan, Long-Term Care insurance, Place of death

## Abstract

**Background:**

Although the Japanese government has expanded its ‘Elderly Housing with Care Services’ (EHCS) to ensure sufficient places of death for the elderly, resident deaths have occurred in less than 30% of the facilities. Our purpose was to identify the factors associated with residents’ deaths in the EHCS, especially within the areas that are expected to have a large increase in the number of deaths.

**Methods:**

Our cross-sectional study involved all EHCS (*N* = 412) in Japan’s Tokyo, Kanagawa prefecture and used self-administered questionnaire data that the EHCS directors completed. In addition, we accessed the national statistics related to the municipal characteristics of the cities where the EHCS were located. These sources provided information about health care provision for the residents as well as facility/resident/regional characteristics that could potentially be associated with residents’ deaths in the EHCS. Based on this information, a sequential multiple logistic regression analysis was performed. First, we included in-facility health care provision (presence of nursing staff) and facility/residents/regional characteristics in Model 1. Next, visiting nurse agency’s care provision was included in Model 2. Finally, we included community hospitals or clinical care provision in Model 3.

**Results:**

One hundred and fifty-four facilities answered the questionnaire (response rate: 37.4%). A total of 114 facilities were analysed. In-facility residents’ deaths occurred in more than half (54.4%) of the facilities. After adjusting for all variables (Model 3), end-of-life (EOL) care provision from community hospitals or clinics, the number of years since establishment and the number of residents were significantly associated with residents’ deaths. In Model 2, visiting nurse’s EOL care provision was significantly associated with residents’ death.

**Conclusion:**

Our results suggest that in order to accommodate residents’ deaths, the government or the facility’s directors should promote the cooperation between EHCS facilities and community hospitals or clinics for in-residents’ EOL care. Furthermore, as the results suggest that community nurses contribute to the occurrences of death by collaborating with the physician, promoting cooperation with visiting nurse agencies may be also needed.

## Background

The population of the world is aging [1]. Japan will be confronted with a large increase in the number of deaths, especially in the capital and adjacent areas [2]. While most of the deaths occur in hospitals [3], the Japanese government does not intend to increase the number of hospital beds to contain elderly healthcare expenditures [4]. This means that the government will have to ensure other places of death.

To ensure sufficient places of death, the government has covered end-of-life (EOL) care services that are provided by home care agencies (e.g. visiting nurse agencies) and aged care facilities (e.g. nursing homes) in the Long-Term Care (LTC) Insurance System. The number of EOL homecare service users is increasing, but it is still difficult for elderly individuals to die at home due to the existing shortage of family caregivers because of the decreasing birth rate. Thus, the demand for aged care facilities where the elderly individuals can be cared for is growing, and this growing demand has led to a lack of nursing home beds [5]. Even so, the government cannot largely increase the number of facilities because institutional care services make high benefit claims to the LTC Insurance System. To ensure that enough beds are available, the government excludes mildly impaired elderly individuals from eligibility for admission to these facilities, but there are still a large number of people waiting for beds (295,000 in 2016) [6].

Such a situation has caused the Japanese government to expand its ‘Elderly Housing with Care Services’ (EHCS). The EHCS is an elderly facility system that was established in 2011 to provide rooms within a familiar environment where the elderly (aged 60 years or over) can live until they die. They are required to provide barrier-free rooms and employ staff who will support residents’ daily living.

The staff are not required to be a physician or nurse. In fact, 60% of the facilities do not employ medical staff. When the residents are in need of health care (including EOL care), they can independently contract with the community hospitals, clinics, or visiting nurse agencies covered by insurance as they are cared for at home. The remaining 40% of the EHCS employ nursing staff as part of their extra services. Some of the facilities contract with the home care agencies to provide additional services, such as health check-ups. In addition, if the residents need further health care, they can receive it from the insurance-covered community home care agencies. In any case, the residents can receive EOL care when they need it.

In contrast to nursing homes, EHCS are not integrated into the LTC Insurance System as part of the institutional care services. Thus, the facility provides the rooms, daily living support, and extra services while not making claims to the LTC Insurance. Instead, the residents pay for it. For this reason, the Japanese government expects that the EHCS will solve the problem of ‘death place crunch of the elderly’ by satisfying ‘the sustainability of LTC Insurance’ and eliminating the ‘difficulty of ensuring family caregivers’. The government introduced subsidies for the establishment, and the EHCS have already established 203,783 rooms [7].

However, in-facility residents’ deaths have occurred in less than 30% of the facilities [8]. If the Japanese government expects EHCS to be one of the death locations, it is necessary to explore the factors associated with residents’ in-facility deaths.

A narrative review reported that it is necessary for nurses to provide EOL health care on a 24-h basis in assisted living (elderly facilities, in the USA) [9]. In Japan, a nationwide survey reported that EHCS where residents had died had higher rates of receiving health care from community medical institutions or visiting nurse agencies than EHCS in which people did not die (e.g. medical institution: death-occurring facilities: 98.0%, death-not occurring facilities: 81.7%) [8]. These findings suggest that health care service availability allows for residents’ deaths in EHCS. However, according to a literature review, the following factors are also associated with residents’ deaths in other aged care facilities: facility characteristics (e.g. confirming preferences for resident’s EOL care and educating the staff concerning EOL care), residents’ characteristics (e.g. older age, female gender, severely dependent for the activities of daily living), and regional characteristics (e.g. hospital bed availability) [10]. To identify the death-related factors of EHCS, it is necessary to also explore, with adjustments, the above characteristics that could potentially be associated with in-facility residents’ deaths.

Therefore, the present study aimed to identify the factors associated with in-facility resident deaths within the EHCS, especially in the areas that are expected to be confronted with a large increase in the number of deaths. The findings will provide useful information to the Japanese policy makers and those of other aging countries, especially those that are introducing the LTC Insurance System.

## Methods

This cross-sectional study used two data sources: a self-administered questionnaire completed by the EHCS directors and publicly-available national statistics related to the regional characteristics of the municipalities where the EHCS facilities were located. This study was approved by the Institutional Review Board of the Tokyo Medical and Dental University (No. 2194).

### Study setting and participants

We conducted a survey involving all EHCS in Tokyo and Kanagawa prefecture, which was adjacent to it. The estimated increase in the number of elderly people from 2010 to 2040 in this area is the highest in the country (projected number of deaths in Tokyo: 1,438,000; Kanagawa: 1,089,000; and Japan (mean of all prefectures): 195,600 [2]); however, the number of beds in hospitals and aged care facilities per number of elderly people are lower than the mean of all the prefectures [3, 11]. The present study enrolled all EHCS in the prefectures (*N* = 412) that were available as of 31 July 2015.

### Data sources and items

#### Questionnaire

We drafted the self-administered questionnaire based on the findings of a previous review on the factors promoting residents’ deaths at aged care facilities and home [10]. Additionally, three directors of the EHCSs that accommodated residents’ death checked the draft questionnaire, and we further revised it according to their advice. The questionnaire included the following items:

1. Residents’ death in the facility
  1. Whether in-facility resident deaths occurred within the EHCS facility since its establishment (death-occurring/death-not occurring). We defined in-facility residents’ deaths as ‘confirmed deaths in the facilities’, and excluded unexpected deaths such as accidental deaths. We did not limit the period of deaths occurring in order to observe the experiences as much as possible, as EHCS was newly introduced in 2011.
  2. The number of residents’ in-facility deaths over the past year. This was a secondary question to confirm whether these facilities actually accommodated residents’ deaths recently. Thus, it was only included in the descriptive analysis.
2. Health care input from community
  1. Receiving care services from visiting nurse agencies (have received care/have not received care); whether visiting nurse agencies have provided EOL care to the residents (yes/no).
  2. Receiving care services from community hospitals or clinics (have received care/have not received care); whether community hospitals or clinics have provided EOL care to the residents (yes/no).
    Regarding receiving care services from community hospitals or clinics/visiting nurse agencies’, we have described the original items that were categorized as ‘have received care’ in Table 1; the original items were only included for the descriptive analysis because this study only sought to examine whether EHCS receive care services from the agencies and not the process of receiving care. In addition, we specifically asked about whether the home care agencies provide EOL care services since visits by physicians or nurses to the EOL population was reported as a strong determinant of home death [12].
3. Facility characteristics
  1. Presence of nursing staff at day/night in the facility (yes/no); there are cases wherein employed nursing staff concurrently serve other facilities. In this case, the staff can be absent from the facility. The present study assessed whether the employed staff were continuously present within the facility because a previous study reported that it is necessary for nurses to provide EOL health care to the residents on a 24-h basis [9]).
  2. Facilities’ efforts related to being the residents’ choices for places of death
    - Confirming preferences for resident’s EOL care with the residents/family members (yes/no).
    - Educating the staff concerning EOL care (yes/no).
  3. Number of years since establishment
4. Resident characteristics
  1. Number of residents
  2. Rate of females
  3. The number of residents categorized into 5-year age groups: In the present study, it was difficult to ask residents’ mean age because the respondents (directors) would have been required to calculate it and the calculating process was predicted to cause calculation error/decreasing response rate. Therefore, they were asked to categorize the number of residents into 5-year age groups, and thereafter, we calculated residents’ mean age using the data and certified it as a variable that reflected residents’ ages. The calculation was performed based on the number of age groups and the mid-point of the groups (60–64: 62; 65–69: 67; 70–74: 72; 75–79: 77; 80–84: 82; 85–89: 87; 90–94: 92; older than 94: 97). For example, if a facility answered ‘5′ in the 60–64 category, we considered it as five residents aged 62 years old.
  4. Rate of residents at each ‘Care Need Level’ in the LTC Insurance System: The Japanese LTC Insurance system categorizes care-needing elderly according to their eligibility level, of which there are seven (Support Required Levels 1–2 and Care Need Levels 1–5; the most severe is Care Need Level 5), according to an elderly person’s physical and cognitive functions. Since the deaths are more likely to be observed in residents with severe impairments, we certified ‘Care Need Levels 3–5 or not’ as a variable that reflects residents’ condition; thus, the rate of residents certified as each levels was only included in the descriptive analysis.
  5. Rate of residents who lived in the municipality where the facility was located: This item was used to confirm whether the death-occurring facilities provided a place of death in a familiar environment for the elderly, according to the purpose of the EHCS.

**Table 1:** The sub items on have received care services from outside the facilities

Response sub items	
Community hospitals or clinics	
Have received care	- Facility contract: Facilities contracting with community hospitals or clinics to provide health care services to residents^a^
	- Resident contract: Residents contracting with community hospitals or clinics to arrange for doctor’s visits for themselves^a^
	- Outpatient: Residents visit community hospitals/clinics to receive outpatient care^a^
Have not received care	- The residents not receiving care from community hospitals or clinics
Visiting nurse agencies	
Have received care	- Facility contract: Facilities contracting with the agencies to provide health care services to residents^b^
	- Resident contract: Residents contracting with the agencies to arrange for a nurse’s visit^b^
Have not received care	- Residents not receiving such services from visiting nurse agencies

^a, b^ multiple answers allowed; if any item was selected, it was considered as ‘have received care’

#### Data collection procedure using the questionnaire

We obtained a list of the facilities from an open website operated by the EHCS registered office [13], and we sent out the questionnaires to these facilities between September and October 2015. To increase the response rate, we contacted each facility’s directors and requested them to cooperate by responding before we sent out the questionnaires, while also taking care to not persuade them too much into responding. After sending out the questionnaires, we visited each cooperating facility to collect the completed questionnaires. Collection of the questionnaires was completed in January 2016.

#### Publicly available data

We confirmed the following regional characteristic data of the municipal location of each EHCS facility from publicly available national statistics [14–17]:

1. Municipal aging rate (the number of people aged 60 years or over/total population). The municipal aging rate represented the number of individuals eligible for EHCS availability in each community.
2. Number of hospital beds/clinics/visiting nurse agencies per 100,000 elderly persons (those aged 60 years or over). The number represented the amount of community health care resources available to the residents.
3. Municipal income level. This was calculated according to municipal taxable/number of taxable individuals. We included this item because the EHCS residents or their family members are required to pay for the complete expenses of their rooms or their daily living support service charges.

### Data analysis

We conducted bivariate analyses following descriptive analyses for all variables. The dependent variable was whether the in-facility resident deaths occurred in EHCS; the remainder of the variables were set as independent. To analyse the dichotomous variables, we used χ^2^ test. When the expected frequencies were <5 in any group, Fisher’s exact test for the comparison of two proportions was used. To analyse the continuous variables, the Wilcoxon rank sum test was used as all the variables were non-normally distributed.

All variables could potentially be associated with residents’ deaths in EHCS [8, 10]. Thus, these were included in the multiple logistic regression model regardless of the association in bivariate analysis following confirmation for multicollinearity using the Variance Inflation Factor (VIF). The VIF was used to determine the effect of collinearity of prediction variables on regression analyses. A VIF > 5 for any two variables was used to indicate collinearity [18].

A sequential logistic regression analysis was used to examine the effect of health care provision to the residents carefully. After examining in-facility health care provision (presence of nursing staff at day/night) and facility/residents/regional characteristics (Model 1), visiting nurse agency’s care provision was included in Model 2. Finally, we included community hospitals or clinical care provision in Model 3. In the multiple logistic regression analyses, we calculated the odds ratios and 95% confidence intervals. The goodness of fit for the model was confirmed using the Hosmer-Lemeshow test. All analyses were performed using SAS version 9.4 software (SAS Institute, Cary, North Carolina, USA).

## Results

### Study sample and basic characteristics

One hundred and fifty-four respondents returned the questionnaires (response rate = 37.4%); however, 40 questionnaires were excluded due to incomplete data. Therefore, the final sample consisted of data from 114 facilities.

In-facility resident deaths occurred in 54.4% of the facilities (Table 2). Among the death-occurring facilities, 75.8% (47/62 facilities) had experienced one or more resident death over the past year. Table 3 shows the basic characteristics of the facility, residents, and regions.

**Table 2:** State of residents’ deaths in EHCS (*n* = 114)

	Number	(Percent)
Death-occurring facility since it was established		
Yes	62	(54.4)
Number of residents’ in-facility deaths over the past year		
0	67	(58.8)
1	16	(14.0)
2	13	(11.4)
3 or more residents	18	(15.8)

*EHCS* Elderly Housing with Care Services

**Table 3:** Basic characteristics of the facility, residents, and regions (*n* = 114)

	n Median	(%) (IQR)
Health care input from community		
Receiving care services from visiting nurse agencies		
Received care	79	(69.3)
[Facility contract]^a^	18	(15.8)
[Resident contract]^a^	68	(59.6)
Visiting nurse agencies provide EOL care to the residents		
Yes	49	(43.0)
Receiving care services from community hospitals or clinics		
Received care	114	(100)
[Facility contract]^b^	50	(43.9)
[Resident contract]^b^	86	(75.4)
[Outpatient]^b^	81	(71.1)
Community hospitals or clinics provide EOL care to the residents		
Yes	71	(62.3)
Facility Characteristics		
Number of years since establishment	3	(2–4)
Presence of day nursing staff		
Yes	31	(27.2)
Presence of night nursing staff		
Yes	2	(1.8)
Confirming residents’ EOL preferences with residents/family members		
Yes	87	(76.3)
Educating staff to help them support resident deaths		
Yes	42	(36.8)
Residents’ Characteristics		
Number of residents	32	(16–44)
Mean age of the residents (years)	83.5	(81.8–84.7)
Rate of females (%)	67.9	(57.1–75.0)
Rate of residents certified as Care Need Levels 3–5 (%)	18.2	(10.5–37.5)
[Rate of residents not requiring care (%)]	11.7	(0.0–24.6)
[Rate of residents certified as Support Required Level 1 (%)]	8.3	(2.3–16.1)
[Rate of residents certified as Support Required Level 2 (%)]	8.0	(2.7–13.6)
[Rate of residents certified as Care Need Level 1 (%)]	21.1	(14.3–28.6)
[Rate of residents certified as Care Need Level 2 (%)]	15.4	(6.8–22.2)
[Rate of residents certified as Care Need Level 3 (%)]	8.8	(3.2–15.6)
[Rate of residents certified as Care Need Level 4 (%)]	5.7	(0.0–13.3)
[Rate of residents certified as Care Need Level 5 (%)]	3.3	(0.0–8.0)
Rate of residents who lived in the same municipality as the facility (%)	46.1	(24.4–61.2)
Regional Characteristics		
Municipal aging rate (aged 60 years or over) (%)	28.8	(28.5–30.4)
Number of hospital beds per 100,000 elderly people (aged 60 years or over)	3082.3	(2426.1–3750.5)
Number of clinics per 100,000 elderly people (aged 60 years or over)	245.9	(204.5–273.1)
Number of visiting nurse agencies per 100,000 elderly people	19.7	(16.7–24.0)
Municipal income level (Yen)^c^	3455.3	(3166.6–3817.8)

*EOL* End-Of-Life, *IQR* interquartile range

^a, b^Multiple answers allowed, ^c^ Municipal income level was calculated according to municipal taxable/number of taxable individuals

### Results of the bivariate analyses

The results of the bivariate analyses are shown in Table 4. The variables with a *p*-value <0.05 were the following: receiving care services from visiting nurse agencies; community hospitals or clinics/visiting nurse agencies providing EOL care to the residents; number of years since establishment; confirming residents’ EOL preferences; educating staff; number of residents; rate of residents certified as Care Need Levels 3–5; rate of residents who lived in the municipality where the facility was located; number of clinics.

**Table 4:** Factors associated with the facilities’ resident deaths: Bivariate analyses (*n* = 114)

		In-facility resident deaths				*p*-value
		death-occurring (*n* = 62)		death-not occurring (*n* = 52)		
		n	%	n	%	
Health care input from community						
Receiving care services from visiting nurse agencies	Have received	49	79.0	30	57.7	0.01^*^
Visiting nurse agencies provide EOL care to the residents	Yes	38	61.3	11	21.2	<0.01^**^
Receiving care services from community hospitals/clinics	Have received	62	100.0	52	100.0	–
Community hospitals or clinics provide EOL care to the residents	Yes	53	85.5	18	34.6	<0.01^**^
Facility Characteristics						
Number of years since establishment	Median (IQR)	3	(3–4)	2.5	(2–4)	<0.01^**a^
Presence of day nursing staff	Yes	17	27.4	14	26.9	0.95
Presence of night nursing staff	Yes	1	1.6	1	1.9	1.00^b^
Confirming residents’ EOL preferences with residents/family members	Yes	56	90.3	31	59.6	<0.01^**^
Educating staff to support resident deaths	Yes	29	46.8	13	25.0	0.02^*^
Residents’ Characteristics						
Number of residents	Median (IQR)	37.5	(24–50)	20	(7.5–33)	<0.01^**a^
Mean age of the residents (years)	Median (IQR)	83.6	(82.3–84.7)	83.2	(81.0–84.6)	0.30^a^
Rate of females (%)	Median (IQR)	67.1	(59.6–74.4)	68.8	(53.8–75.0)	0.61^a^
Rate of residents certified as Care Need Levels 3–5 (%)	Median (IQR)	24.6	(15.0–38.8)	15.4	(5.8–33.3)	0.02^*a^
Rate of residents who lived in the same municipality as the facility (%)	Median (IQR)	36.8	(19.4–58.8)	49.2	(33.3–70.4)	0.02^*a^
Regional Characteristics						
Municipal aging rate (%)	Median (IQR)	29.3	(28.5–30.4)	28.7	(28.1–30.1)	0.07^a^
Number of hospital beds per 100,000 elderly (aged 60 years or over)	Median (IQR)	3098.2	(2045.1–3950.6)	2674.8	(2426.3–3275.0)	0.75^a^
Number of clinics per 100,000 elderly people (aged 60 years or over)	Median (IQR)	228.2	(204.5–268.4)	256.8	(214.8–273.1)	0.03^a^
Number of visiting nurse agencies per 100,000 elderly people	Median (IQR)	18.5	(15.8–21.3)	19.8	(17.5–24.0)	0.06^a^
Municipal income level (Yen)	Median (IQR)	3392.9	(3166.6–3766.2)	3587.9	(3281.8–3817.8)	0.10^a^

*EOL* End-Of-Life, *IQR* interquartile range

^a^Wilcoxon rank sum test. Other variables: χ^2^ test or ^b^Fisher’s exact test

^*^ *p* < 0.05, ^**^ *p* < 0.01

### Factors associated with in-facility resident deaths

Before conducting the sequential multiple logistic regression analysis, we excluded ‘receiving care services from community hospitals or clinics’ from the analysis because all the facilities had received it. In addition, ‘municipal income level’ was also excluded because the variable had VIF scores of 14.2, along with ‘number of clinics’ (all the other variables had VIF scores under 3).

The results of the sequential multiple logistic regression analysis are shown in Table 5. Confirming residents’ EOL preferences was significantly associated with in-facility death in the Model 1. After including visiting nurse agency’s care provision, the EOL care provision was significantly associated with residents’ death (Model 2). After adjusting for all the variables, EOL care provision from community hospitals or clinics was significantly associated with facilities’ in-facility resident deaths (Model 3). Other than that, the number of residents and the number of years since establishment were positively associated with residents’ death in all of the Models.

**Table 5:** Factors associated with residents’ deaths: Multiple logistic regression analysis (*n* = 114)

		Model 1		Model 2		Model 3	
		OR	95% CI	OR	95% CI	OR	95% CI
Health care input from community							
Receiving care services from visiting nurse agencies	Have received			2.50	0.52–11.9	5.32	0.75–37.5
Visiting nurse agencies provide EOL care to the residents	Yes			4.24	1.11–16.2	1.13	0.20–6.29
Hospitals or clinics provide EOL care to the residents	Yes					14.0	2.51–78.1
Facility Characteristics							
Number of years since establishment	Unit = 1 year	2.07	1.28–3.37	2.55	1.42–4.60	2.92	1.50–5.68
Presence of day nursing staff	Yes	0.38	0.11–1.29	0.68	0.17–2.68	0.73	0.15–3.51
Presence of night nursing staff	Yes	12.5	0.40–390.9	4.52	0.10–205.2	1.54	0.03–70.4
Confirming residents’ EOL preferences with residents/family members	Yes	5.44	1.16–25.5	2.42	0.44–13.4	1.85	0.26–13.1
Educating staff to support resident deaths	Yes	1.40	0.42–4.69	1.36	0.35–5.40	0.65	0.13–3.31
Residents’ Characteristics							
Number of residents	Unit = 5	1.27	1.08–1.50	1.25	1.05–1.49	1.23	1.02–1.48
Mean age of residents (years)	Unit = 5	1.22	0.37–3.97	1.31	0.34–5.03	2.01	0.47–8.63
Rate of females (%)	Unit = 5%	1.00	0.83–1.22	1.02	0.82–1.27	1.02	0.81–1.29
Rate of residents certified as Care Need Levels 3–5 (%)	Unit = 5%	1.03	0.88–1.20	1.05	0.88–1.25	1.06	0.87–1.28
Rate of residents who lived in the same municipality as the facility (%)	Unit = 5%	0.94	0.84–1.05	0.95	0.84–1.06	0.95	0.83–1.08
Regional Characteristics							
Municipal aging rate (%)	Unit = 5%	1.64	0.56–4.83	1.58	0.53–4.70	1.51	0.45–5.09
Number of hospital beds per 100,000 elderly people (aged 60 years or over)	Unit = 100	0.98	0.94–1.02	1.00	0.95–1.04	1.00	0.95–1.05
Number of clinics per 100,000 elderly people (aged 60 years or over)	Unit = 10	1.01	0.94–1.10	0.99	0.89–1.10	0.99	0.88–1.12
Number of visiting nurse agencies per 100,000 elderly people (aged 60 years or over)	Unit = 1	0.93	0.83–1.05	0.94	0.83–1.06	0.94	0.82–1.08
Hosmer-Lemeshow test	*p*-value	0.15		0.41		0.18	

*EOL* End-Of-Life

## Discussion

The present study clarified the following determinants of deaths occurring in EHCS: EOL care provision from community hospitals or clinics, higher number of residents, and a long period since the facility’s establishment.

Even after adjusting for relevant variables, EOL care service provision from community hospitals or clinics had a considerable impact on in-facility death. This result is consistent with the findings of studies on other aged care facilities [19–22]; the findings indicated that physician’s or the palliative care team’s EOL care provision in the facilities were associated with in-facility residents’ death. A previous study reported that EOL care provision contributes to reducing hospitalization and improving pain management in the facilities [23]. Furthermore, the involvement of a physician who can provide care makes it possible to issue a death certificate in the facility. These effects of EOL care provision may contribute to accommodating deaths in EHCSs. It may be essential to receive the EOL care from community hospitals or clinics because the facility is not required to employ a physician. To accommodate resident’s death in EHCS, policy makers or directors of the facilities should promote systematic collaboration between EHCS and the medical institutions.

Although a previous study has suggested an association between visiting nurse agency’s care service provision and residents’ deaths in EHCS [8], this association was found only in Model 2 and not in Model 3, adjusting for the involvement of community hospitals or clinics diminished this association. In the Japanese health care system, community nurses provide most of the care services according to the instructions of the primary physician. It is assumed that the present results represent this system. However, the result suggests that although community nurses’ EOL care provision was not a determinant of the residents’ deaths, nurses play a role in accommodating in-facility deaths by collaborating with the physician.

On the other hand, the presence of nursing staff in EHCS was not associated with the in-facility death in any of the Models. In Japan, over 80% of the visiting nurse agencies provide nursing services on a 24-h basis [24]. However, a previous nationwide survey reported that about 6% of the EHCSs employed nursing staff for night-shifts [25] (in the present study, it was about only 1.8%, especially in Tokyo and Kanagawa prefectures). Considering this, the difference in the effects may be due to whether they can provide care at all hours. This inference is consistent with a suggestion by a previous study that it is necessary for nurses to provide EOL health care at elderly facilities on a 24-h basis [9]. According to a previous study, general practitioners (GPs) often have limited time to be with the older patients [26]. To accommodate in-facility deaths, health care providers who can provide the care at all hours based on the instruction of physician are necessary. Although the presence of nurses at all hours or cooperation with visiting nurse agencies is needed, it may be difficult to increase the number of EHCSs that employ nurses because it is not currently a requirement to employ nurses. To support the deaths occurring in EHCSs, policy makers or facility directors need to develop a system to promote cooperation with visiting nurse agencies for 24-h-based visits.

Confirming residents’ EOL preferences was a factor among the facility characteristics that was found to affect in-facility death. This association was found only in the Model 1, wherein health care input from community was not adjusted for. The results suggest that the community health care agencies were involved in the confirmation process or that the confirmation may have led to the agencies providing care. Further studies are needed to examine when this confirmation occurs or who does it.

The ‘number of residents’ and the ‘number of years since establishment’ were positively associated with in-facility deaths. The facilities that have these characteristics may have more opportunities of resident deaths. In addition, these facilities may have the know-how or manpower to support the residents in EOL than other facilities. However, it is assumed that these characteristics had an impact on in-facility deaths as EHCS was newly introduced in 2011. The effect will change as time passes or if the cooperation between EHCS and community health care agencies is further enhanced.

Finally, the present findings will be useful for other areas of Japan as well as other countries that will be facing similar aging situations soon, especially countries that have introduced the LTC Insurance System (e.g. Korea). If sufficient places of death for the elderly while limiting insurance expenditures need to be ensured, a system should be developed that allows for physicians’ visits to the residents in EOL of EHCS or a similar aged care facility. In addition, to support EOL care provision, it is needed to establish a system to promote cooperation with visiting nurse agencies at all hours.

## Limitations

A few limitations of the present study should be acknowledged. First, the response rate was low. It may have affected the percentage of facilities where deaths occurred (it may have been easier for the death-occurring facilities to respond). In addition, the present Models might have statistical problems such as type 1 error and relative bias [27] due to the small sample size. However, the results are not inconsistent with the previous findings and the current Japanese health care system. This study provides useful information for discussion on how sufficient places of death can be ensured.

Second, the present study utilised the occurrence of in-facility resident deaths since its establishment as the outcome measure. This measure might not reflect the current situation of in-facility deaths. However, the answer has a certain amount of confidence underlying it because 75.8% of the death-occurring facilities responded that they had actually experienced deaths of the residents over the past year.

Third, we calculated the mean age of the residents using the number of age groups and the mid-point of the groups instead of each resident’s age. However, the calculated age was almost same as the previous nationwide survey’s result [8] (the present study [median of the calculated age]: 83.5 years old; previous survey [median of the residents’ age]: 83.0 years old).

Finally, residents’ deaths cannot only be evaluated by measuring death quantities. Future studies will need to provide more knowledge about high-quality EOL care in EHCS.

## Conclusions

We sought to identify the factors associated with deaths in EHCS and found that health care provision, especially in EOL care, from community hospitals or clinics have a strong effect on the deaths occurring in the facility. To ensure sufficient places of death in Japan, policy makers or facility directors should promote cooperation between the medical institution and EHCSs. Furthermore, to support the EOL care provision in hospitals or clinics, developing a system to promote cooperation with visiting nurse agencies at all hours is also required.

## Abbreviations

EHCS: Elderly Housing with Care Services
EOL: End-of-life
GP: General practitioner
LTC: Long-Term Care
VIF: Variance Inflation Factor
